# Removal of the A_10_ adenosine in a DNA-stabilized Ag_16_ nanocluster†

**DOI:** 10.1039/d0ra02672g

**Published:** 2020-06-23

**Authors:** Cecilia Cerretani, Jiro Kondo, Tom Vosch

**Affiliations:** Nanoscience Center and Department of Chemistry, University of Copenhagen Universitetsparken 5 Copenhagen 2100 Denmark tom@chem.ku.dk; Department of Materials and Life Sciences, Sophia University 7-1 Kioi-cho, Chiyoda-ku 102-8554 Tokyo Japan j.kondo@sophia.ac.jp

## Abstract

The role of the terminal adenosine (A_10_) on the spectroscopic and structural properties of a previously described DNA-stabilized Ag_16_ nanocluster (DNA:Ag_16_NC) is presented. In the original DNA:Ag_16_NCs (5′-CACCTAGCGA-3′), the A_10_ nucleobase was involved in an Ag^+^-mediated interaction with an A_10_ in a neighboring asymmetric unit, and did not interact with the Ag_16_NC. Therefore, we synthesized AgNCs embedded in the corresponding 9-base sequence in order to investigate the crystal structure of these new DNA-A_10_:Ag_16_NCs and analyze the photophysical properties of the solution and crystalline state. The X-ray crystallography and spectroscopic measurements revealed that the 3′-end adenosine has little importance with respect to the photophysics and structure of the Ag_16_NCs. Additionally, the new crystallographic data was recorded with higher spatial resolution leading to a more detailed insight in the interactions between the nucleotides and Ag atoms.

## Abstract 2

We investigated the effect of removing the A_10_ from 5′-CACCTAGCGA-3′ on the photophysical and structural properties of a DNA-stabilized Ag_16_NC.

## Graphical abstract

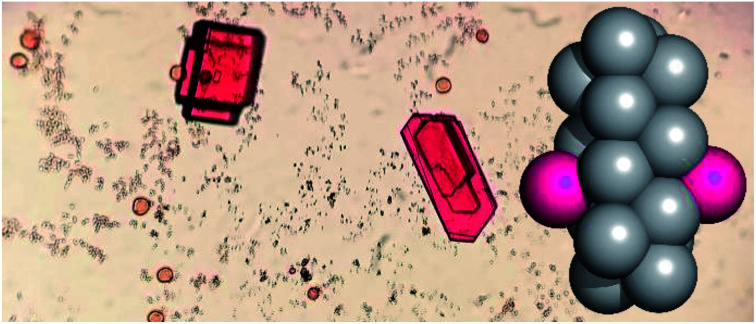

## Introduction

DNA-stabilized silver nanoclusters (DNA:AgNCs) are a new and intriguing class of fluorophores that contain a limited number of silver atoms (usually < 30) wrapped in one or several single stranded DNA oligomers.^1–8^ These emitters have been finding uses for diverse applications spanning from sensing to fluorescence imaging.^9–12^ The color, brightness and photostability of AgNCs formed in the DNA scaffold are difficult to predict based on the DNA sequence, although significant advances have been made using machine learning tools.^13–15^ In addition, atomic structures of AgNCs and their interactions with the DNA scaffold have started to appear recently in the literature.^16,17^ In our previous work, we have presented a DNA:AgNC composed of two 10-base DNA strands (5′-CACCTAGCGA-3′) and a Ag_16_ cluster (further referred to as DNA:Ag_16_NC).^17^ This 5′-CACCTAGCGA-3′ sequence originated from a large data set developed by Copp *et al.*^14^ Intriguing aspects of DNA:Ag_16_NCs comprise the unusually large Stokes shift of about 5600 cm^−1^ and the near-infrared emission, peaking at 736 nm. These properties make them interesting fluorescent probes given the high transparency of biological material in this wavelength range. Further details on the photophysical properties and the crystal structure of DNA:Ag_16_NCs can be found in previous publications.^17,18^ Besides the Ag_16_ core, the crystal structure of DNA:Ag_16_NCs also revealed the presence of three Ag positions with an occupancy below one. One of these three Ag positions is a cation that mediates an interaction between two terminal adenosines (designated A_10_) of different asymmetric units in the crystal.^17^ The A_10_ nucleotide is not involved in any direct interaction with the Ag_16_NC. An open question was whether these three silver positions with lower than 1 occupancy were three docking positions of the same atom or separate atoms. Additionally, we postulated that the silver cation coordinating two terminal A_10_ nucleotides had no influence on the photophysical properties of the Ag_16_NC. To address these questions, we synthesized DNA:AgNCs using the sequence 5′-CACCTAGCG-3′, where the terminal A_10_ was not present (further referred to as DNA-A_10_:Ag_16_NCs). Prior to single crystal X-ray diffraction measurements, the HPLC-purified DNA-A_10_:Ag_16_NCs were investigated in solution by steady-state and time-resolved fluorescence spectroscopy. Additionally, emission spectra and fluorescence decay times were also measured for several DNA-A_10_:Ag_16_NC crystals in order to confirm that the photophysical properties in the crystalline state are similar to the solution state.

## Results and discussion

### Photophysical properties of DNA-A_10_:Ag_16_NCs in solution

**Fig. 1:**
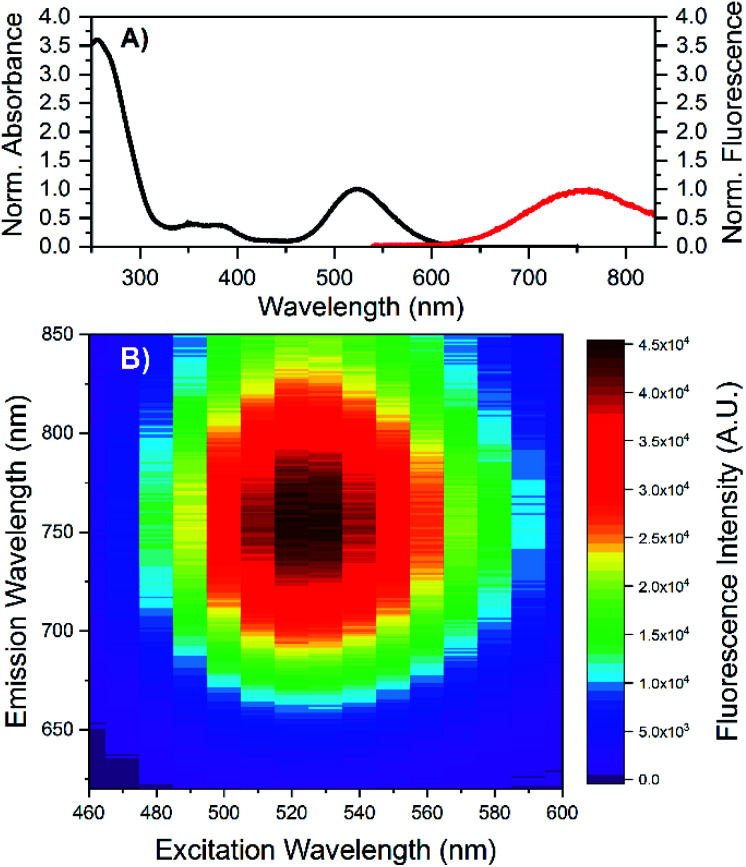
(A) Normalized absorption and steady-state emission of DNA-A_10_:Ag_16_NCs in 10 mM NH_4_OAc. The emission spectrum was acquired by exciting the sample at 507 nm (Picoquant, LDH-P-C-510). (B) Steady-state 2D emission *versus* excitation plot of DNA-A_10_:Ag_16_NCs in 10 mM NH_4_OAc. All measurements were performed at room temperature.

HPLC allowed us to collect a purified fraction around 18 minutes that displayed absorbance at 530 nm and emission at 730 nm (see Fig. S1†). The steady-state and time-resolved fluorescence properties of this fraction were then studied in a 10 mM ammonium acetate (NH_4_OAc) solution. The removal of the terminal A_10_ has no significant effect on the absorption spectrum in the visible range (see Fig. S2†), whereas the DNA absorption around 260 nm is slightly lower compared to the original DNA:Ag_16_NCs since there is one less nucleotide in the DNA sequence. The emission spectrum of DNA-A_10_:Ag_16_NCs in Fig. 1A displays an even larger Stokes shift than the original DNA:Ag_16_NCs.^18^ As a result, a Stokes shift of 5893 cm^−1^ is found at room temperature between the absorption maximum at 523 nm and the emission maximum of 756 nm (Fig. 1A). The 2D emission *versus* excitation plot in Fig. 1B shows the presence of a well-defined emitter and no shift of the emission maximum with increasing excitation wavelength.^19^ The fluorescence quantum yield of DNA-A_10_:Ag_16_NCs was determined to be 0.26 (see Fig. S3†) and is comparable to that of DNA:Ag_16_NCs.^18^ Based on the steady-state findings, we can conclude that a similar emitter is formed using 5′-CACCTAGCGA-3′ or 5′-CACCTAGCG-3′ strands. The removal of the terminal adenosine mainly causes a red shift of the emission spectrum, but does not affect the absorption and fluorescence quantum yield.

**Fig. 2:**
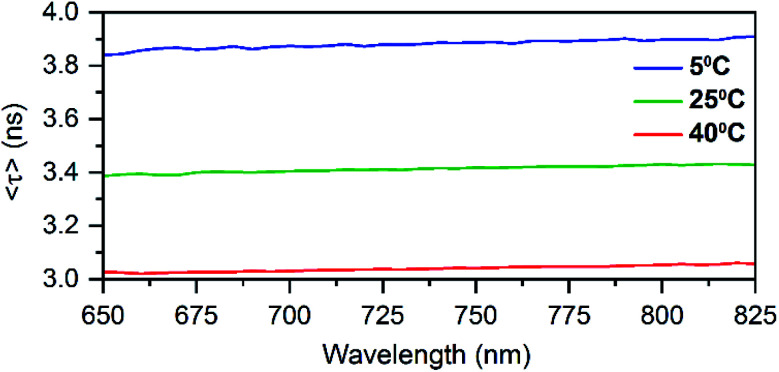
Average decay time spectra of DNA-A_10_:Ag_16_NCs in 10 mM NH_4_OAc at different temperatures.

Time-correlated single photon counting experiments were performed at various temperatures (5 °C, 25 °C and 40 °C), and the average fluorescence decay times, weighted over the whole emission spectrum,^18,20^ can be found in Table 1. These values are again very similar to those of DNA:Ag_16_NCs with a maximum deviation of 0.2 ns. Reconstructing Time-Resolved Emission Spectra (TRES, see Fig. S4†) shows that the spectral relaxation for DNA-A_10_:Ag_16_NCs in 10 mM NH_4_OAc occurs predominantly on a time-scale below the instrument response function (IRF, 150 ps), similarly to DNA:Ag_16_NCs.^18^ This is not always the case, and a number of DNA:AgNCs with slow spectral relaxation on the time scale of the fluorescence decay time have been reported before.^20–22^ As a result of this faster spectral relaxation, the average decay time is fairly constant over the whole emission range (see Fig. 2).^19^

**Table 1:** Absorption and emission maxima, Stokes shift, weighted average fluorescence decay time (<*τ*_w_>) and fluorescence quantum yield (*Q*) at different temperatures

	5 °C	25 °C	40 °C	RT
*λ*_abs_ (max)	—	—	—	523 nm
*λ*_em_ (max)	—	—	—	756 nm
Stokes shift	—	—	—	5893 cm^−1^
<*τ*_w_>^a^	3.88 ns	3.42 ns	3.04 ns	—
*Q* b	—	—	—	0.26

a Average decay time, weighted by the intensity over the whole emission range. For further details, see ESI Table S2.

b Cresyl violet in ethanol (*Q* = 0.56) was used as reference dye.^24^ The horizontal line indicates that this data was not measured.

In principle, if there is no slow spectral relaxation at all, the fluorescence decay time for the DNA:AgNCs should become mono-exponential. Table S2† shows that a single exponential reconvolution model gives similar decay times as tail-fitting the decays with a single exponential, but with poorer reduced *χ*^2^ values. This clearly indicates that the presented average decay times can be considered as a single exponential decay with minimal distortion by spectral relaxation (mainly on the time scale close to the IRF time scale).^18,20^ The average decay time also drops linearly with increasing temperature similar to DNA:Ag_16_NC and the red-emitting DNA:AgNC stabilized by 5′-TTCCCACCCACCCCGGCCC-3′.^18,20^

In addition, time-resolved anisotropy measurements at different temperatures were carried out in order to determine the average hydrodynamic volume (*V*_hydro_) of DNA-A_10_:Ag_16_NCs. A *V*_hydro_ of 9.72 nm^3^ was found (see Fig. S5†) using the Perrin equation.^23^ This value is similar but slightly smaller than the *V*_hydro_ of 10.5 nm^3^ obtained for DNA:Ag_16_NCs.^18^

Based on steady-state and time-resolved finding in solution, we can already confidently conclude that the removal of the terminal A_10_ has no significant impact on the photophysical properties of the cluster, besides a small red shift in the emission spectrum.

### Photophysical properties of DNA-A_10_:Ag_16_NC crystals

**Fig. 3:**
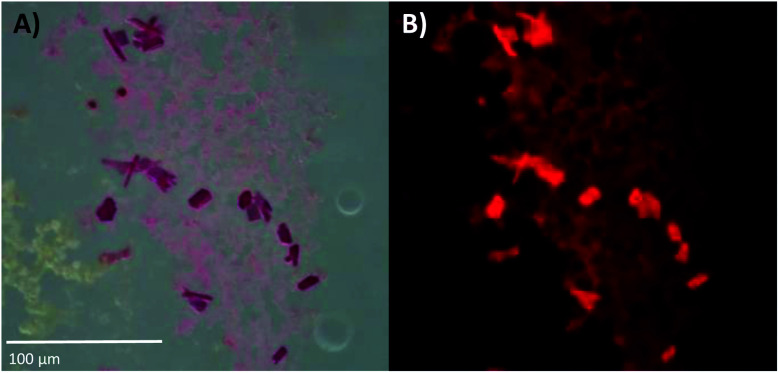
(A) Bright-field image of DNA-A_10_:Ag_16_NC crystals, recorded with a color camera. (B) Wide-field fluorescence image of the crystals in (A), excited with green light (510–550 nm). The blue edge of the NIR emission extends into the red region of the spectrum, thus the crystals appear to fluoresce red on a color camera. The pictures of these crystals were acquired about three weeks after preparing the crystallization droplet. See Fig. S6† for a picture of the entire crystallization well.

DNA-A_10_:Ag_16_NCs were crystallized by the hanging-drop vapor-diffusion method using 10% 2-methyl-2,4-pentanediol (MPD), 10 mM spermine and 200 mM Ca(NO_3_)_2_ in 50 mM 3-(*N*-morpholino)propanesulfonic acid (MOPS) buffer (pH = 7). This condition differs from the previously reported DNA:Ag_16_NCs,^17^ where a lower concentration of Ca(NO_3_)_2_ (100 mM) and PEG 3350, rather than MPD, were used. Fig. S6 and S7,† respectively, show images of DNA-A_10_:Ag_16_NC and DNA:Ag_16_NC crystals acquired under the same bright field and fluorescence conditions, in order to highlight similarities and differences. The bright field image in Fig. 3A displays 10–20 μm sized crystals surrounded by precipitation. The fluorescence images in Fig. 3B confirms the NIR emission (red on a color camera) upon excitation with green light. It also proves that the magenta precipitate surrounding the crystals contains the same emissive DNA-A_10_:Ag_16_NCs. The crystals analyzed by X-ray diffraction were obtained from the same HPLC-purified solution, but were grown in a different well-plate.

**Fig. 4:**
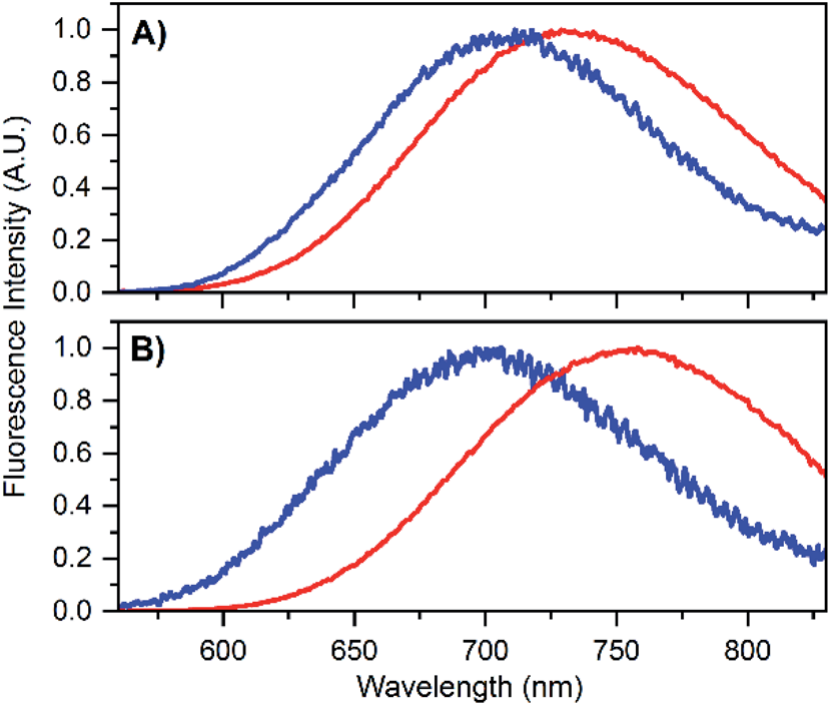
(A) Emission spectrum of DNA:Ag_16_NCs in 10 mM NH_4_OAc solution (red) and in crystalline state (blue). The solution spectrum is taken from Bogh *et al.*,^18^ while the crystal spectrum is from Cerretani *et al.*^17^ Additional spectra of DNA:Ag_16_NC crystals can be found in the ESI† of Cerretani *et al.*^17^ (B) Emission spectrum of DNA-A_10_:Ag_16_NCs in 10 mM NH_4_OAc solution (red) and in crystalline state (blue). Emission spectra of different crystals can be found in Fig. S8.†

To test that the crystallized emitters have a similar structure to those in solution, we measured emission spectra and recorded fluorescence decay times of different crystals (see Fig. S8 and S9†). The emission spectra of the DNA-A_10_:Ag_16_NC crystals are blue-shifted with respect to the solution, featuring an emission maximum close to 700 nm instead of 756 nm as in solution (see Fig. 4B). A blue shift of the crystal emission spectrum was also observed for the original DNA:Ag_16_NC, which had a solution maxima of 736 nm (see Fig. 4A). Interestingly, it seems that the removal of the terminal A_10_ nucleotide red-shifts the solution spectrum but yields a similar crystal spectrum in comparison to the original DNA:Ag_16_NC. We speculate that the difference in the emission maxima in solution might be related to the conformational flexibility of DNA, and that the removal of the terminal A_10_ nucleotide allows the AgNC to achieve a larger spectral relaxation in solution. In the crystalline state, the spectra are alike and this is in agreement with the fact that the crystal structures are isomorphous (see below).

The blue shift in the crystal spectra of DNA-A_10_:Ag_16_NCs and DNA:Ag_16_NCs might indicate that the crystal packing limits the range of spectral relaxation achievable in both cases. Fluorescent decay times recorded from five different crystals of DNA-A_10_:Ag_16_NCs yielded similar average decay times (see Fig. S9†) and an average value of 2.79 ns was calculated. This is approximately 1 ns shorter than the solution value (see Table 1), but still surprisingly unquenched for such a densely packed array of emitters. For example, it was shown recently that dried red-emitting DNA:AgNCs display heavily quenched fluorescence with a decay time that is more than 8 times shorter than the solution value.^25^ Despite the blue shift and faster decay time of the DNA-A_10_:Ag_16_NCs in the crystallized form, it is fair to say that the crystal structure represents a state that is largely similar to the solution structure.

### Crystal structure of DNA-A_10_:Ag_16_NCs

**Fig. 5:**
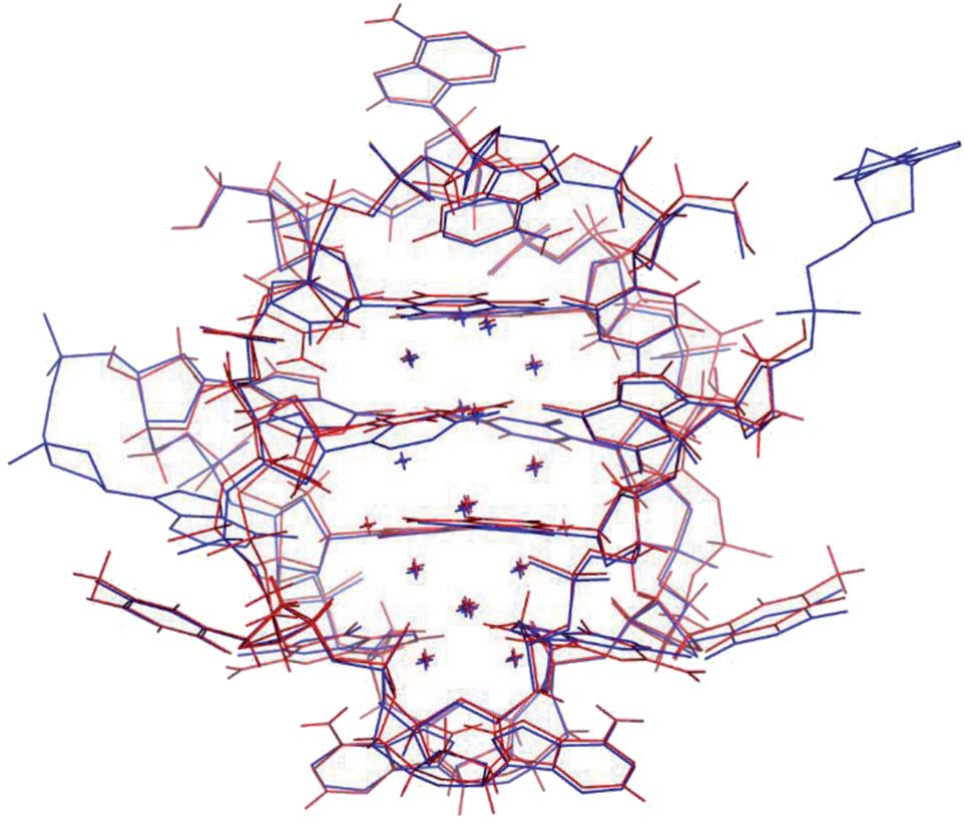
Overlay of a subunit structure of DNA:Ag_16_NC (blue) and DNA-A_10_:Ag_16_NC crystals (red). The asymmetric unit cell consists of two subunits. The crosses indicate the positions of Ag atoms.

**Fig. 6:**
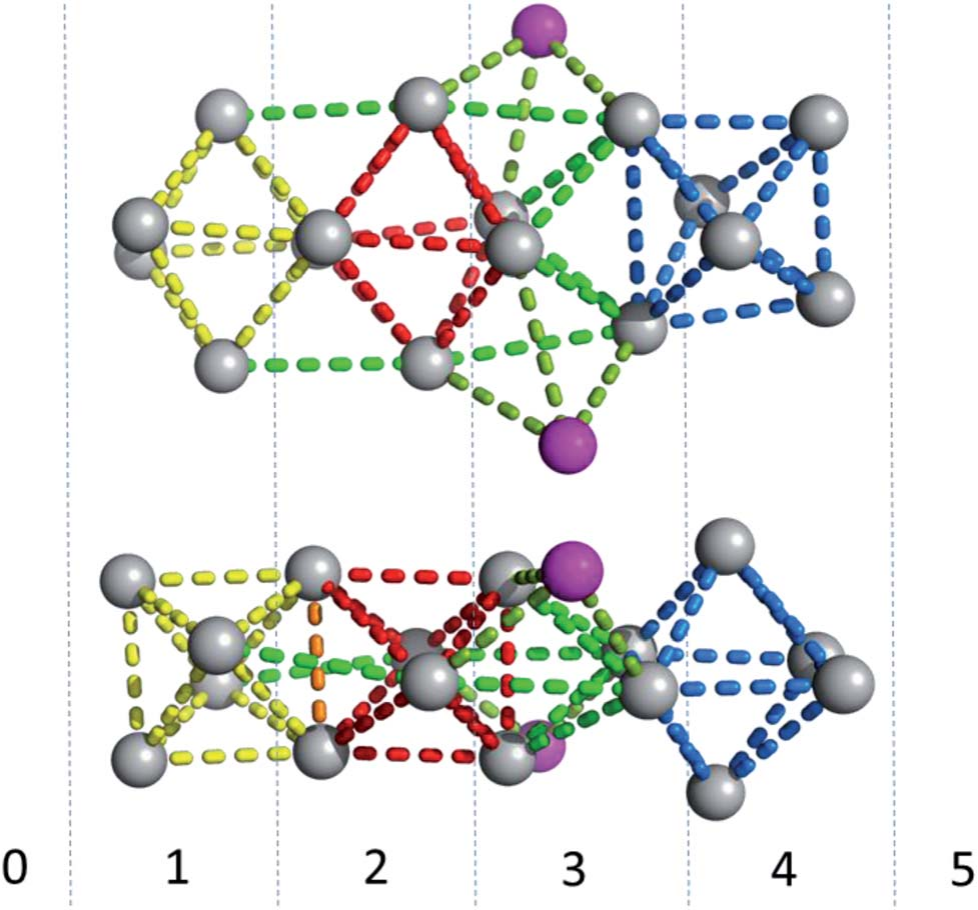
Side- and top-view of the Ag_16_NC (grey spheres) of DNA-A_10_:Ag_16_NCs. The magenta spheres represent the silver atoms with low occupancy (around 0.3). Red, blue and yellow lines are drawn between silver atoms to create octahedrons for displaying purposes. The other Ag–Ag interactions are given by light and darker green lines. The Ag_16_NC is divided in six artificial sections, indicated with numbers from 0 to 5.

**Fig. 7:**
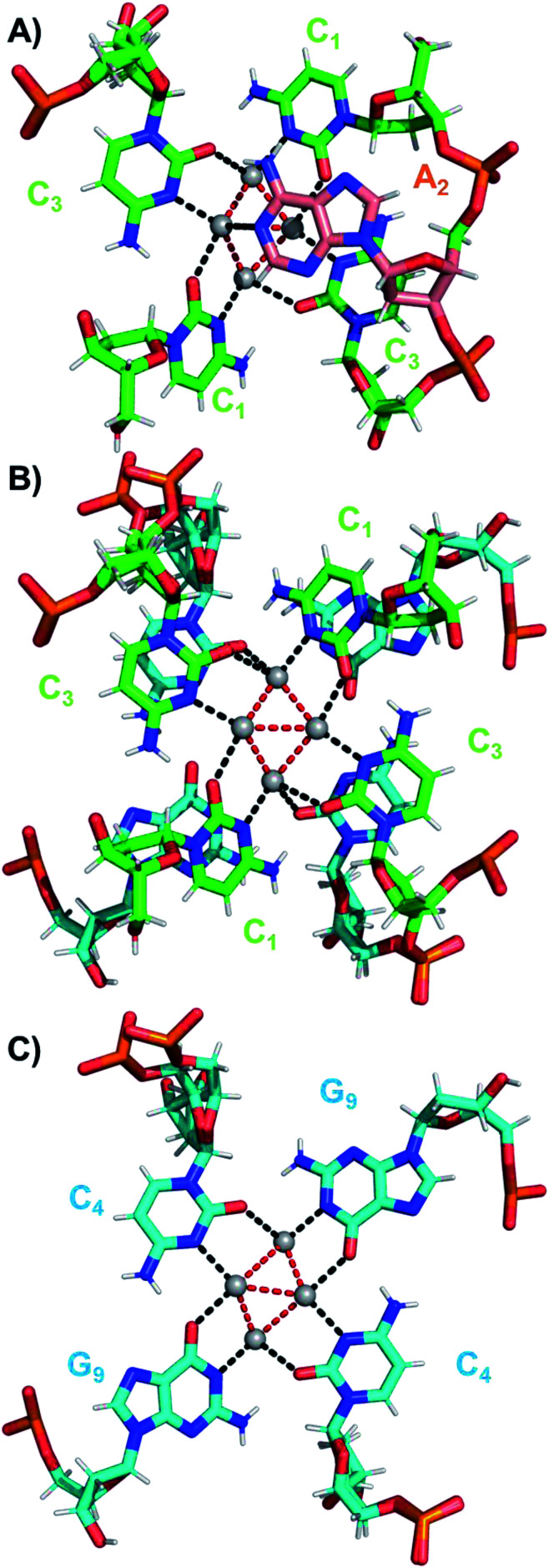
(A) Sections 0 and 1, (B) section 1 and a part of section 2, and (C) section 2 of the DNA-A_10_:Ag_16_NC subunit. Red dashed lines indicate Ag–Ag interactions, while black dashed lines represent coordination bonds.

**Fig. 8:**
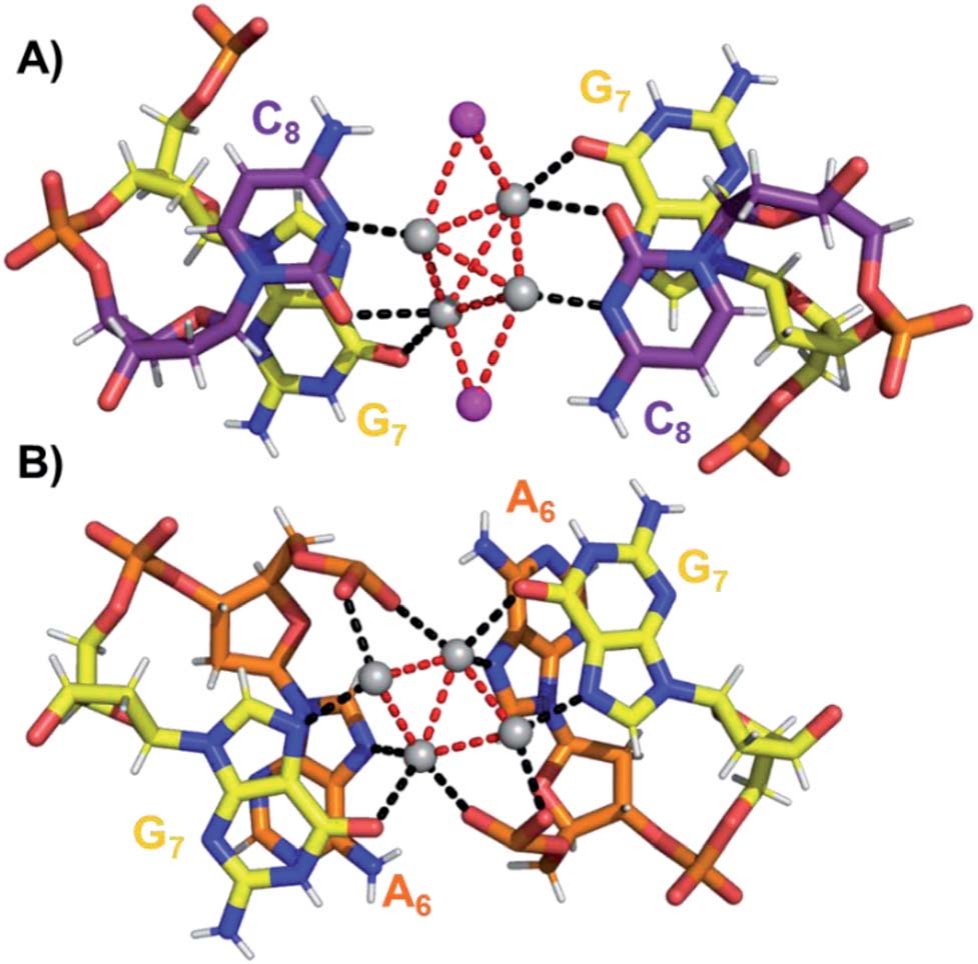
(A) Section 3, and (B) sections 4 and 5 of the DNA-A_10_:Ag_16_NC. Red dashed lines indicate Ag–Ag interactions, whereas black lines represent coordination bonds.

The photophysical characterization of the DNA-A_10_:Ag_16_NC solution and crystals hinted at structural similarities with the DNA:Ag_16_NC. Fig. 5 shows the overlay of a subunit of the asymmetric unit cell for the DNA:Ag_16_NC (blue) and DNA-A_10_:Ag_16_NC crystals (red). Clearly, the overall structure is very similar and the removal of the terminal A_10_ does not influence significantly the arrangement of silver atoms within the core of the cluster. However, the removal of A_10_ results in the lack of the Ag^+^ cation interacting with A_10_ nucleotides of different asymmetric units in the original DNA:Ag_16_NCs (see PDB database, accession code 6JR4). The structural isomorphism can also be deduced by the crystallographic values shown in Table S3.† DNA-A_10_:Ag_16_NC has indeed the same space group *P*2_1_, similar unit cell parameters and the presence of two subunits in the asymmetric unit. This also indicates that the A_10_–Ag^+^–A_10_ interaction, observed in the original DNA:Ag_16_NCs, was not very critical for the asymmetric units' packing in the crystal structure. Each subunit in the asymmetric unit consists out of two DNA oligonucleotides, 16 Ag atoms (grey spheres) and two silver positions with low occupancy (around 0.3). The latter are represented by magenta spheres in Fig. 6, 7 and 8.

Fig. 6 gives a representation of the Ag positions in the DNA-A_10_:Ag_16_NC. A way to visualize the arrangement of Ag atoms is by using octahedrons (yellow, red and blue dashed lines). The Ag_16_NC can be described by three distorted octahedrons: two share an edge and the third one is 90° tilted with respect to the first two. The octahedrons do not represent all the Ag–Ag interactions in the Ag_16_NC. Additional Ag–Ag interactions are displayed by green lines (see Fig. 6). The exact charge of the Ag_16_NC is not known at this point, but previous results using mass-spectroscopy on other DNA:AgNCs indicate partially oxidized AgNCs.^26–28^ Based on the Ag_16_NC, we divided the subunit in six artificial sections (see Fig. 6). Details on the structure of DNA-A_10_:Ag_16_NC can be found at the PDB database using accession code 6M2P.

Here we limit ourselves to describing Ag–Ag interactions and the Ag coordination bonds to the nucleotides. Ag–Ag interactions are indicated for distances that are below the sum of the van der Waals radii (3.44 Å). These interactions range from 2.6 to 3.2 Å and a histogram of the distances can be found in Fig. S10.†

The coordination bonds are defined as one interaction of an aromatic nitrogen to the nearest Ag atom and up to two interactions of a carbonyl or phosphate oxygen with the nearest Ag atoms. The coordination bonds described here range from 2.2 to 2.8 Å and a histogram of the distances can be found in Fig. S10.†Fig. 7A and B show the four Ag atoms of section 1. These four Ag atoms interact with five nucleotides: two C_1_s and two C_3_s *via* N3 and O2, and one A_2_*via* N1. Additionally, two Ag atoms can also interact with two C_4_s *via* O2 (Fig. 7B). Fig. 7C displays the next four silver atoms from section 2 that form coordination bonds with two G_9_s *via* N1 and O6 and with two C_4_s *via* N3 and O2. The third section is depicted in Fig. 8A. This region, unlike the rest of the structure, contains six Ag positions; four Ag atoms interact with two C_8_s *via* N3 and O2 and two G_7_s *via* O6, whereas the other two silver positions (magenta spheres) are only bound to Ag atoms, but not to the nucleotides. The fact that they do not interact with a nucleotide could be the reason for the occupancy below 1. These two Ag atoms could potentially move more freely inside the structure, resulting in a lower occupancy compared to the other nucleotide-bound Ag atoms. Fig. 8B displays sections four and five. The four Ag atoms of section 4 interact with two G_7_s *via* N7 and O6, and two A_6_s through N7 and the oxygen atoms of the phosphate groups.

As stated above, the crystal structures of DNA-A_10_:Ag_16_NC and DNA:Ag_16_NC share many similarities that can explain their comparable photophysical properties. However, the latest X-ray data was collected with 1 Å wavelength, and a 1.1 Å resolution was obtained. At this resolution, hydrogen atoms are generally added in the structure refinement and this was also done in this case.

Moreover, we can confirm that G_9_ (Fig. 7C) is deprotonated at the N1 position, and a hydrogen bonding network together with π–π stacking interactions ensures the stability of the entire complex (see PDB database, accession code 6M2P). The increased spatial resolution allowed us to detect alternative conformations (especially of phosphate groups, see Fig. 7). The absence of the A_10_ has no major impact on the crystal formation although crystallization conditions were slightly different. The C_4_, A_6_ and C_8_ take a C3′-*endo* conformation. In one of two strands, the terminal G_9_ also takes C3′-*endo*. Other residues take a C2′-*endo* sugar pucker, which is the general conformation in the B-form DNA duplex. Furthermore, thymine interactions between subunits and Ca^2+^ ions still facilitate crystal packing (see PDB database, accession code 6M2P).

## Experimental

### Steady-state absorption and emission spectroscopy

The absorption measurements were carried out on a Cary 300 UV-vis spectrophotometer (Agilent Technologies). Steady-state fluorescence measurements were performed using a FluoTime300 instrument (PicoQuant) with a 507.5 nm pulsed laser (LDH-P-C-510) or a Xenon arc lamp for the steady-state 2D emission *versus* excitation plot. All fluorescence spectra were corrected for the wavelength dependency of the detector systems, and the 2D map was also corrected for the Xe lamp power.

### Time-correlated single photon counting

Time-resolved fluorescence and anisotropy measurements were performed using a FluoTime300 instrument from PicoQuant with a 507.5 nm pulsed laser (LDH-P-C-510) as excitation source.

### Acquisition and analysis of TRES data

Time-resolved emission spectra (TRES) were acquired by increasing the emission monochromator in steps of 5 nm, from 650 to 825 nm, with an integration time of 60 s per decay in order to achieve at least 10 000 counts in the maximum at the emission maximum. The analysis of time-resolved data was performed with Fluofit v.4.6 from PicoQuant. All decays were first fitted globally with a mono- and bi-exponential reconvolution model including scattered light contribution and the IRF (instrument response function), and then analyzed by tail-fit with 1 exponent, resulting in the same decay time values (see Table S2†). The obtained TRES were corrected for the detector efficiency and transformed to wavenumber units by multiplying with the Jacobian factor (10^7^/*ν*^2^).^29^ TRES were interpolated with a spline function using the built-in *spaps* MATLAB function with a tolerance of 10^−10^ (forcing the interpolated curve to go through the data points). The curve was interpolated using wavenumber steps equivalent to 0.001 nm wavelength steps. The emission maxima were taken as the maxima of the interpolated TRES. The average decay time <*τ*> of every decay was calculated as the intensity-weighted average. The intensity-weighted decay time <*τ*_ω_> was calculated as the average of <*τ*> over the emission spectra weighted by the steady-state intensity.^19^

### Acquisition and analysis of time-resolved anisotropy data

Time-resolved anisotropy measurements were carried out by exciting the sample with vertically polarized light at 507.5 nm (LDH-P-C-510) and acquiring both vertically and horizontally polarized fluorescence intensity decays. The decays were fitted by Fluofit v.4.6 from PicoQuant. A tri-exponential and a mono-exponential reconvolution model were used, respectively, for the decay time and the rotational correlation time (*θ*), including the IRF. The Perrin equation^23^*θ* = *ηV*_hydro_/*k*_B_*T*, where *η* is the dynamic viscosity of the solvent, *V*_hydro_ is the hydrodynamic volume of the species and *k*_B_*T* is the product between the Boltzmann constant (*k*_B_) and the absolute temperature (*T*), allowed us to calculate the hydrodynamic volume of the DNA-A_10_:Ag_16_NCs. For simplicity, the Perrin model assumes that the investigated species is spherical. Time-resolved fluorescence and anisotropy measurements were performed at three different temperatures: 5 °C, 25 °C and 40 °C.

### DNA-A_10_:Ag_16_NC synthesis and crystal growth

The silver nanoclusters used in this work are stabilized by the shorter version of the DNA strand reported by Bogh *et al.*,^18^ therefore the resulting DNA-A_10_:Ag_16_NCs were synthesized and HPLC-purified the same way. Details can be found in the ESI.† After HPLC purification, the sample was solvent-exchanged several times by spin-filtration (Amicon Ultra-2 Centrifugal Filter Unit with 3 kDa cut-off membrane) into 10 mM NH_4_OAc in order to remove any free silver cations and improve the chemical stability over time.

Crystals were grown in an incubator at 20 °C by the hanging-drop vapor-diffusion method. 1 μL of cluster solution (approx. 330 μM concentration) was mixed with 1 μL of crystallization buffer and equilibrated against 250 μL of 40% 2-methyl-2,4-pentanediol (MPD). The crystals formed in the presence of 10% MPD, 10 mM spermine, 50 mM 3-(*N*-morpholino)propanesulfonic acid (MOPS) with pH = 7 and 200 mM Ca(NO_3_)_2_. A crystal was scooped by a nylon cryoloop (Hampton Research) and then flash-frozen in liquid nitrogen prior to the X-ray experiment.

### X-ray data collection

X-ray data was collected at 100 K with synchrotron radiation at the BL-5A beamline in the Photon Factory (Tsukuba, Japan). A 1 Å X-ray beam, the default wavelength in the BL-5A beamline, was chosen for the data collection. Pictures of the mounted crystal before and after the experiment can be found in Fig. S11.† The data set was collected using 1° oscillation with 0.5 s exposure per frame. No significant radiation damage was observed when comparing the first and last diffraction image, see Fig. S12.†^30^

### Structure determination and refinement

The data set was processed by the program XDS.^31^ The initial phase was determined with AutoMR from the Phenix suite^32,33^ by molecular replacement using the DNA:Ag_16_NC structure as a model (PDB-ID = 6JR4). A molecular model was constructed by using the program Coot.^34,35^

In the asymmetric unit of the crystal, two Ca^2+^ ions were found. The Ca^2+^ ions were easily distinguished from Ag by the height of their electron density maximum and coordination structures. The atomic parameters were refined by using the program phenix.refine of the Phenix suite.^32^ The final structural resolution was 1.1 Å. Due to the atomic resolution, hydrogen atoms were included in the structure refinement and anisotropic b-factors were applied for all atoms except from the hydrogen atoms.

The atomic coordinate and experimental data have been deposited in the Protein Data Bank (PDB) with the accession code 6M2P.

## Conclusions

In this paper we showed that removal of the terminal A_10_ had no significant impact on the photophysical and structural properties of the Ag_16_NC. Both DNA-A_10_:Ag_16_NC and DNA:Ag_16_NC form isomorphous crystals with asymmetric unit cells of similar dimensions. The increased spatial resolution allowed us to confirm the deprotonation of the G_9_ residues and identify alternative conformations (mainly sugar and phosphate backbone related) in the crystal structure, which could not be observed in the previous DNA:Ag_16_NCs due to the lower spatial resolution. The crystal structure, together with the photophysical characterization contributes also to a more general understanding of ligand-stabilized silver clusters.^36,37^ The DNA-A_10_:Ag_16_NC has one less Ag position with occupancy below 1, which indicates that this Ag^+^ cation plays no significant role with respect to the absorption properties of the Ag_16_NC and its removal does not prevent crystallization. The emission maximum of DNA-A_10_:Ag_16_NCs (756 nm) in solution is red-shifted with respect to DNA:Ag_16_NCs (736 nm), which might be linked to the removal of the A_10_.

## Conflicts of interest

There are no conflicts to declare.

## Supplementary Material

RA-010-D0RA02672G-s001
