# Supplementary material for: Removal of the A10 adenosine in a DNA-stabilized Ag16 nanocluster
Source: RSC Adv. 2020 Jun 23;10(40):23854–60. doi: 10.1039/d0ra02672g (PMC9054913; doi:10.1039/d0ra02672g)
Supplement: RA-010-D0RA02672G-s001 [file RA-010-D0RA02672G-s001.pdf]

Electronic Supplementary Material (ESI) for RSC Advances.
This journal is © The Royal Society of Chemistry 2020

Supporting Information

Removal of the A_{10} adenosine in a DNA-stabilized Ag_{16} nanocluster.

Cecilia Cerretani,[a] Jiro Kondo[*,b] and Tom Vosch[*,a]

Nanoscience Center and Department of Chemistry, University of Copenhagen, Universitetsparken 5, 2100 Copenhagen, Denmark. Email: tom@chem.ku.dk

Department of Materials and Life Sciences, Sophia University 102-8554, Tokyo, Japan. E-mail: j.kondo@sophia.ac.jp

Detailed information about the synthesis and HPLC purification of DNA-A_{10}:Ag_{16}NCs.

The DNA-A_{10}:Ag_{16}NCs were synthesized by mixing hydrated DNA (5'-CACCTAGCG-3', IDT, standard desalting) with $AgNO_3$ (≥99.998%, Sigma Aldrich) in a 10 mM ammonium acetate (NH_4OAc) solution (pH 7.0) prepared in nuclease-free water (IDT). After 15 min, the sample was reduced by 0.5 equivalents of $NaBH_4$ (99.99%, Sigma Aldrich) per silver cation. The final ratio of [DNA]:[Ag^+]:[BH_4^-] was 25 μM : 187.5 μM : 93.75 μM. The sample was kept in the fridge for three days prior to HPLC purification. After purification, the solvent was exchanged to 10 mM NH_4OAc by spin-filtration (cut-off = 3 kDa) since DNA:AgNCs have a high stability in this medium.

The HPLC purification was performed using a preparative HPLC system from Agilent Technologies with an Agilent Technologies 1260 infinity fluorescence detector, an Agilent Technologies 1100 Series UV-Vis detector, and a Kinetex C18 column (5 μm, 100 Å, 250 x 4.6 mm). The mobile phase was a gradient mixture of 35 mM triethylammonium acetate (TEAA) in Milli-Q water and methanol. The elution gradient is described in Table S1.

Table S1. Elution gradient for HPLC purification of DNA-A_{10}:Ag_{16}NCs.

Time (min)	% 35 mM TEAA in MeOH
0-2	15%-20%
2-22	20%-40%
22-24	40%-95%

The run was followed by 6 min of washing with 95% 35 mM TEAA in methanol to remove any remaining sample from the column. The flow rate was 1.3 mL/min.
As shown by the chromatogram in Figure S1, a pure fraction of DNA-A_{10}:Ag_{16}NCs was collected at ~36% 35 mM TEAA in MeOH (around 18 min) by using the absorbance signal at 530 nm.

Quantum yield (Q) determination

The fluorescence quantum yield was determined by a relative method, using a Cresyl Violet in absolute ethanol (Q_R = 0.56) as reference.[1] The absorption and emission spectra were recorded at different concentrations for the sample and the reference. The integrated emission spectra were then plotted as a function of the fraction of absorbed light at the excitation wavelength ($f = 1 - 10^{-A}$). The data was fitted linearly while fixing the y-intercept at zero, and the slopes were used to calculate the quantum yield based on the following equation:

$$Q_S = Q_R \cdot \left(\frac{\alpha_S}{\alpha_R}\right) \cdot \left(\frac{n_S^2}{n_R^2}\right)$$

where *S* and *R* stand for the sample and reference, respectively, *Q* is the quantum yield, α is the slope of the linear regression and *n* is the refractive index of the solvent.

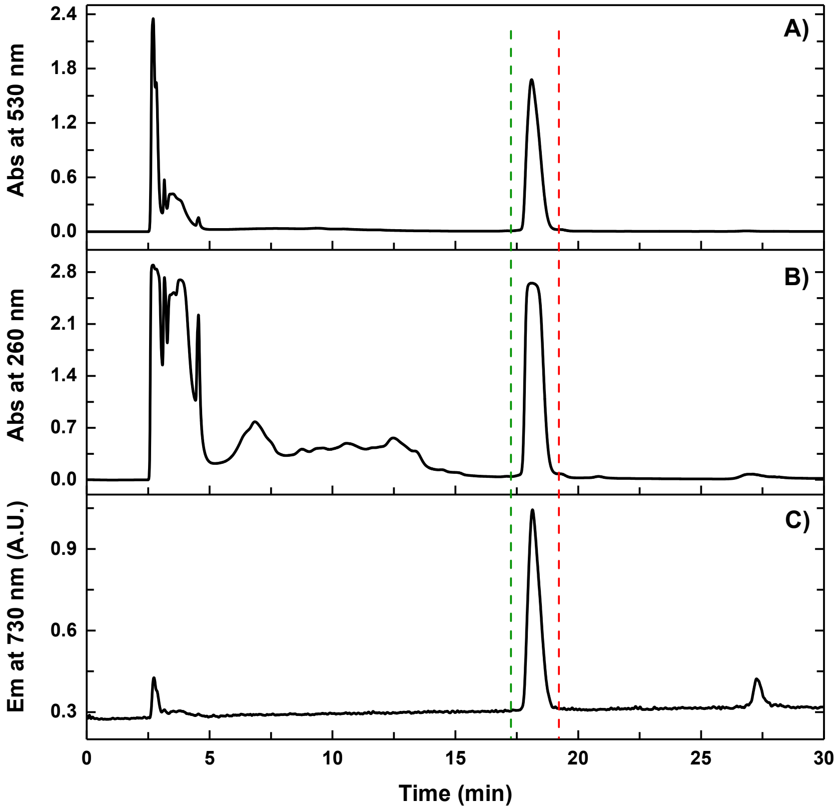

Figure S1: HPLC chromatograms of the NIR-emitting DNA-A_{10}:Ag_{16}NCs. **A)** Absorption chromatogram measured at 530 nm monitoring the absorbance of silver nanoclusters. **B)** Absorption chromatogram measured at 260 nm monitoring the DNA absorbance. **C)** Fluorescence chromatogram measured at 730 nm (λ_{exc}=530 nm) monitoring the DNA-A_{10}:Ag_{16}NCs emission. The purified fraction was collected around 18 minutes (~36% TEAA in methanol). See also reference 2 for further details.

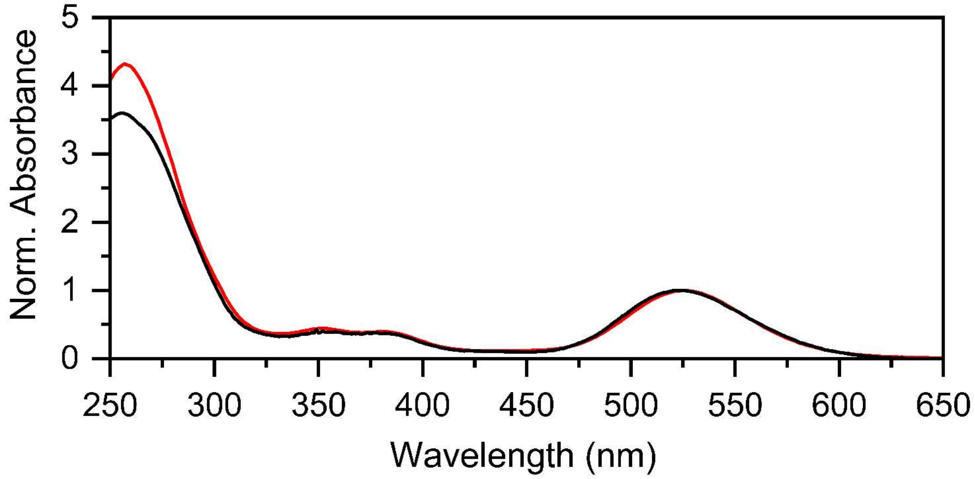

Figure S2: Normalized absorption spectra of DNA:Ag_{16}NCs (red) and DNA-A_{10}:Ag_{16}NCs (black). Data for the red curve was taken from reference 2.

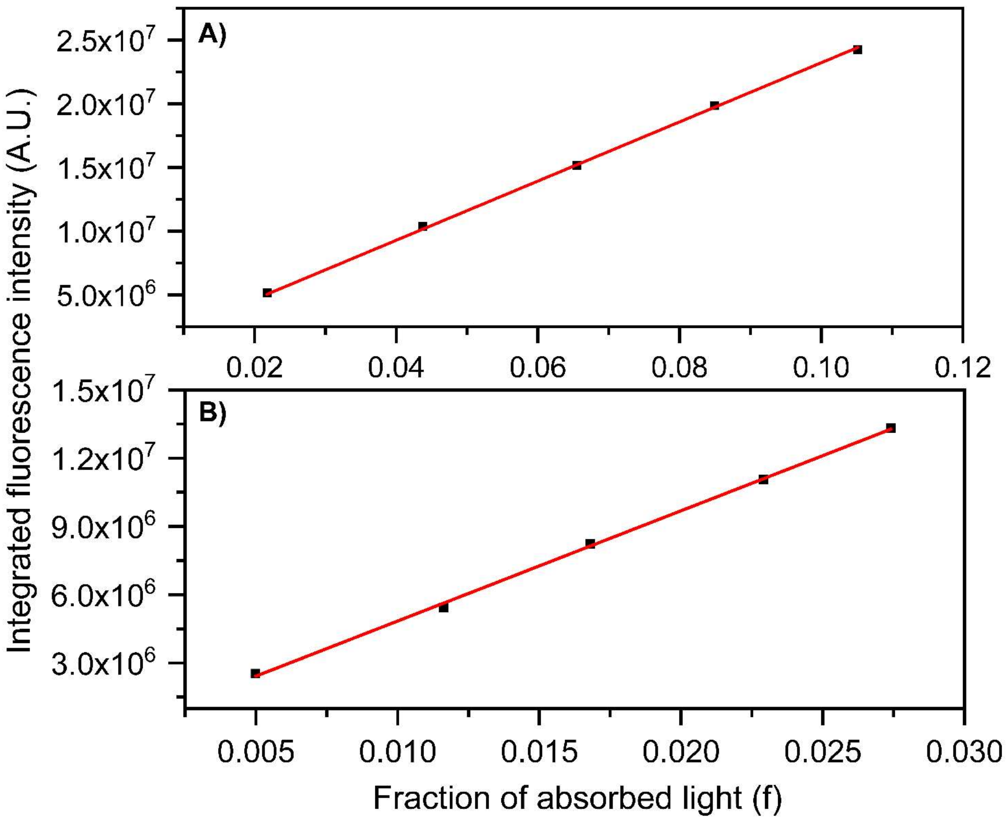

Figure S3. Linear fits of the integrated fluorescence counts plotted as a function of the absorbed light fraction for **A)** DNA-A_{10}:Ag_{16}NCs in 10 mM NH_4OAc and **B)** Cresyl Violet in absolute ethanol. The slopes of the fits (2.32×10^8 and 4.84×10^8 for the DNA-A_{10}:Ag_{16}NCs and reference dye, respectively) were used to determine the fluorescence quantum yield of the DNA-A_{10}:Ag_{16}NCs. The fraction of absorbed light is defined as $f = 1\text{-}10^{-A}$, where A is the absorbance at the excitation wavelength.

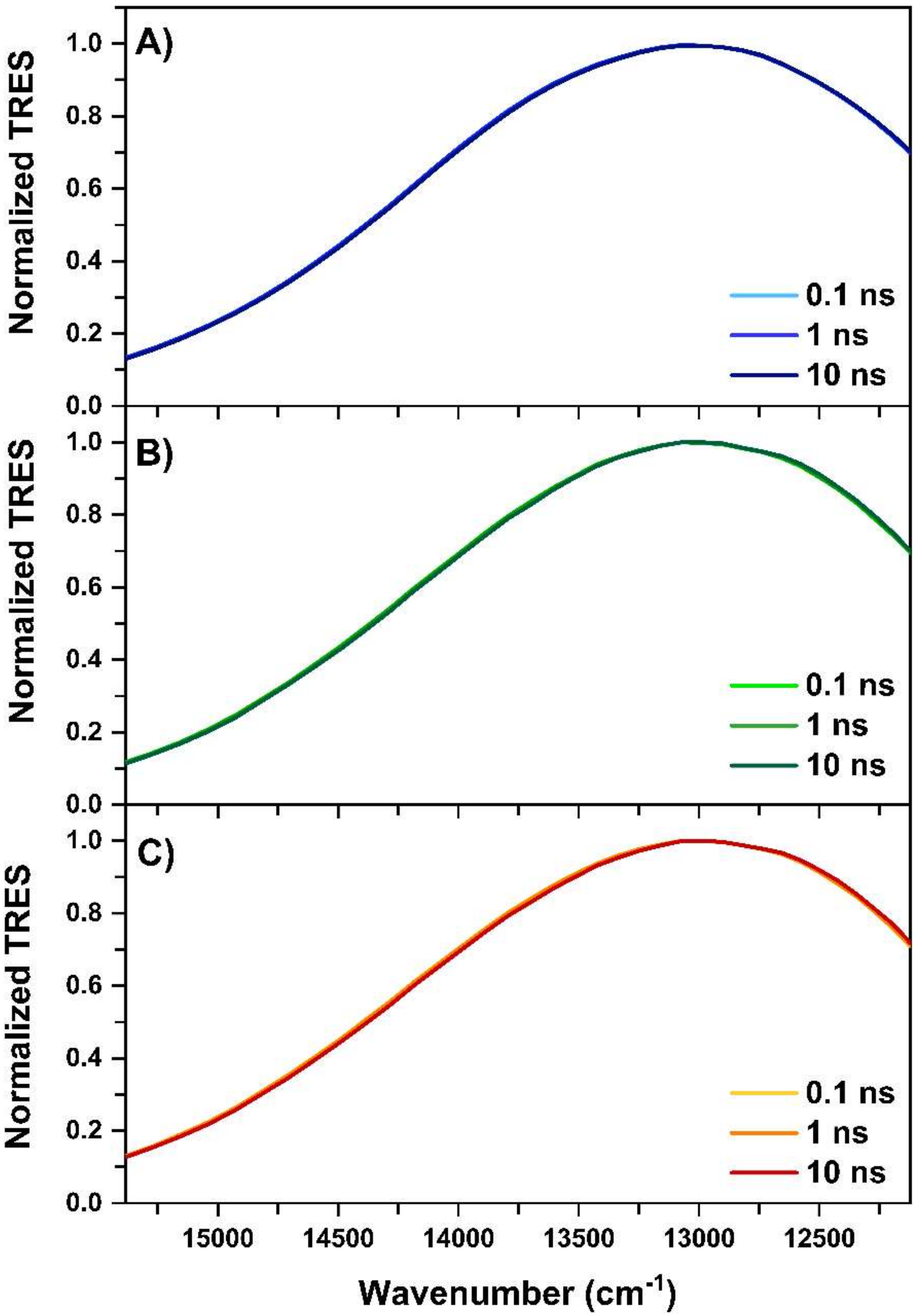

Figure S4. Time-resolved emission spectra (TRES) of DNA-A_{10}:Ag_{16}NCs in 10 mM NH_4OAc at different temperatures: **A)** 5ºC, **B)** 25ºC and **C)** 40ºC.

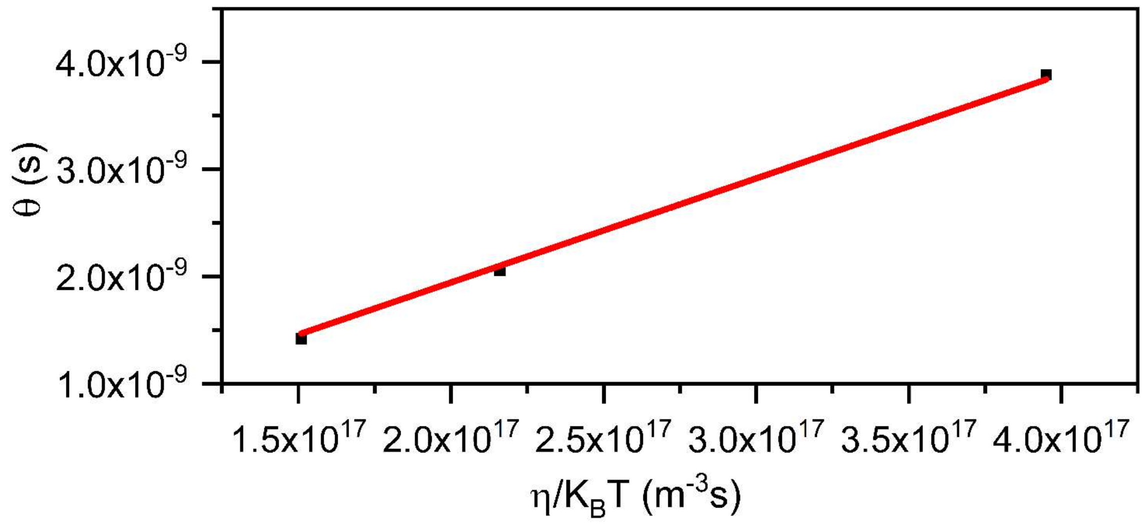

Figure S5. Linear fit of rotational correlation times (θ) as a function of η/k_BT for DNA-A_{10}:Ag_{16}NCs. The intercept is fixed at zero, and the slope represents the hydrodynamic volume.

TCSPC fits.

Table S2. Decay time and anisotropy fits of DNA-A_{10}:Ag_{16}NCs in 10 mM NH_4OAc solution at different temperatures: 5°C, 25°C and 40°C. $\mathbf{I_i}$ is the fractional intensity of every decay time $\boldsymbol{\tau_i}$ at the emission maximum, **θ** is the rotational correlation time and $\boldsymbol{\langle\tau_w\rangle}$ is the intensity-weighted average decay time weighted over the whole emission range.

	T	# exp	τ_1 (ns)	I_1	τ_2 (ns)	I_2	$\langle\tau_w\rangle$	χ^2
**Magic Angle (Global Data Fit)**	**5°C**	**1 exp.**	3.86	100%	-	-	3.86	1.17
		**2 exp.**	4.96	15.97%	3.67	84.03%	3.88	1.09
		**Tail fit**	3.88	100%	-	-	3.88	1.01
	**25°C**	**1 exp.**	3.41	100%	-	-	3.41	1.49
		**2 exp.**	3.51	92.41%	2.22	7.59%	3.42	1.13
		**Tail fit**	3.44	100%	-	-	3.44	1.03
	**40°C**	**1 exp.**	3.03	100%	-	-	3.03	2.09
		**2 exp.**	3.14	92.61%	1.81	7.39%	3.04	1.18
		**Tail fit**	3.09	100%	-	-	3.09	1.04
**Anisotropy**	**T**	**# exp**	**θ (ns)**	χ^2				
	**5°C**	**3 exp.**	3.88	1.02				
	**25°C**	**3 exp.**	2.05	1.09				
	**40°C**	**3 exp.**	1.42	1.16				

Spectroscopic characterization of DNA-A_{10}:Ag_{16}NC and DNA:Ag_{16}NC crystals. Bright-field and fluorescence images were recorded on an inverted Olympus IX71 equipped with an Olympus CPlanFL N 10x objective. For bright-field images, a white LED source creating a white halo of light on top of the sample and a 30:70 beam splitter (XF122 Omega Optical) as dichroic were used. For fluorescence images, an X-Cite Series 120Q light source was used in combination with an Olympus BP510-550 excitation filter, Olympus BA590 emission filter and Semrock FF580-FDi01 dichroic filter. All images in Figure 3 and Figures S6 and S7 were acquired with a SONY XPERIA XZ mobile phone camera.

Confocal spectra from individual DNA-A_{10}:Ag_{16}NC crystals were recorded on an inverted confocal microscope (Olympus IX71) equipped with an Olympus CPlanFL N 10x objective. 507.5 nm pulsed laser (LDH-P-C-510, Picoquant) was used as excitation source (10 MHz, 3 nW on top of the sample) in combination with an excitation filter (Semrock FF02-510/10-25) and an emission filter (Semrock BLP01-532R-25). The dichroic filter cube consisted of an Olympus BP510-550 excitation filter, Olympus BA590 emission filter and Semrock FF580-FDi01 dichroic filter. The emission spectra were recorded with a spectrometer (Princeton Instruments SPEC-10:100B/LN_eXcelon CCD camera with SP 2356 polychromator, 300 grooves/mm). X-axis calibration was performed using the emission lines of a neon spectral lamp (6032 Newport), while y-axis calibration was done by measuring a reference spectrum on an intensity-calibrated Fluotime300 (PicoQuant). Emission spectra (Figure S8) were acquired with 5 s integration time.

Fluorescence decay time measurements were performed on the same inverted confocal microscope (Olympus IX71) equipped with an Olympus CPlanFL N 10x objective. 507.5 nm pulsed laser (LDH-P-C-510, Picoquant) was used as excitation source (10 MHz, 0.3 nW on top of the sample) in combination with the same excitation, emission and dichroic filters employed for recording the emission spectra. The fluorescence signal was detected by an avalanche photodiode (Perkin-Elmer CD3226) connected to a single-photon counting module (Becker & Hickl SPC-830).

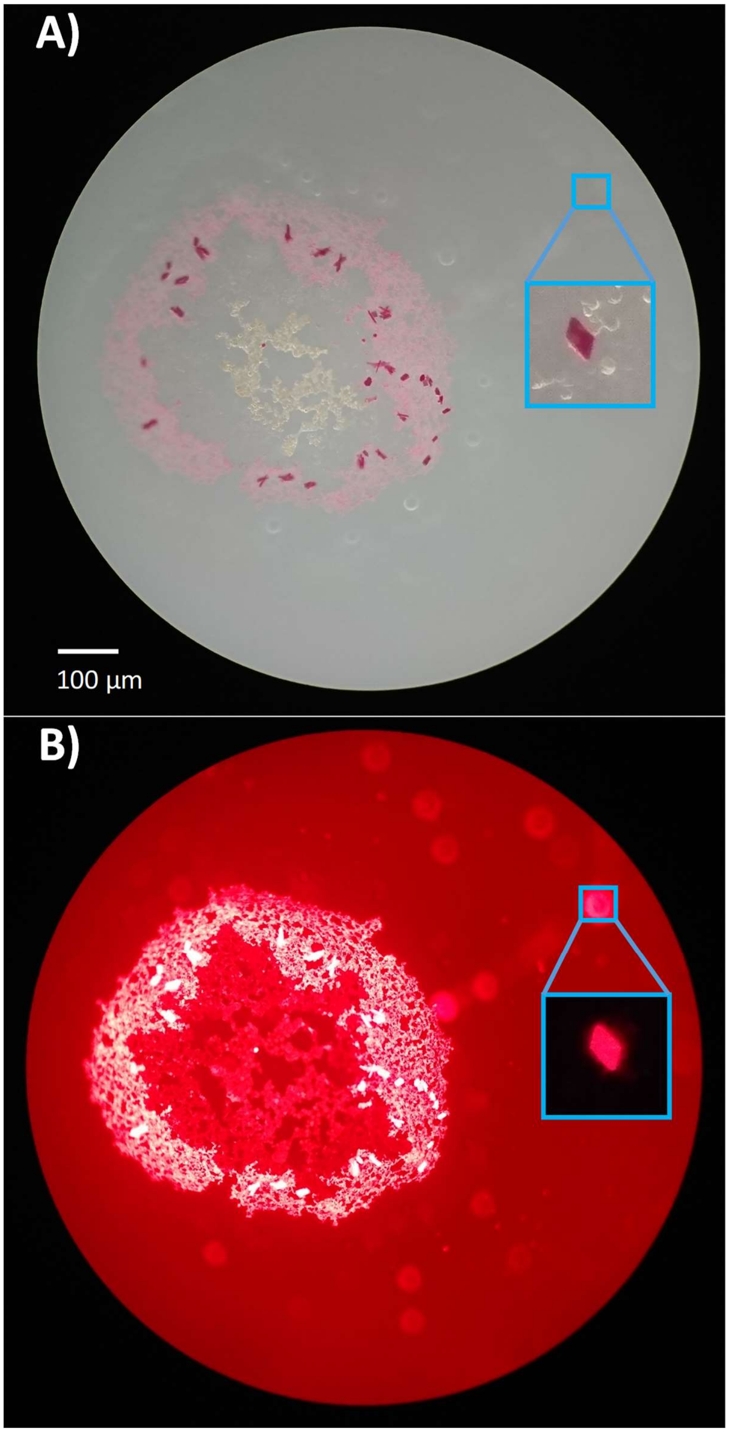

Figure S6: DNA-A_{10}:Ag_{16}NC crystals. A) Bright field image of a crystallization well at 10x magnification. The inset at 40x magnification shows an isolated crystal at a different focal plane. **B)** Fluorescence image of the same region as Figure S6A. The fluorescence intensity of the crystals saturated the intensity of the camera (white color). The inset shows the fluorescence of an isolated crystal with lower exposure time. Images were taken about three weeks after starting the crystallization.

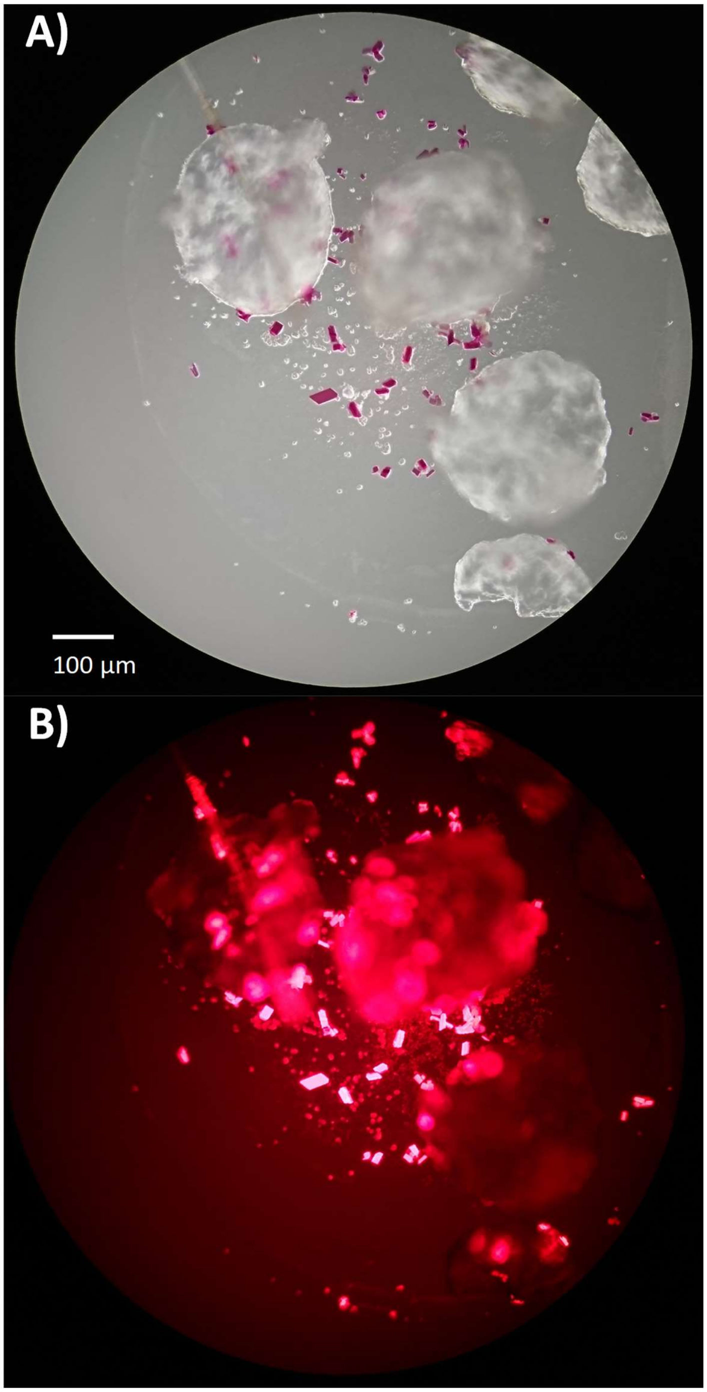

Figure S7: DNA:Ag_{16}NC crystals. A) Bright field image of a crystallization well at 10x magnification. **B)** Fluorescence image of the same region as Figure S7A. The fluorescence intensity of the crystals saturated the intensity of the camera (white color). Images were taken approximately 4 months after starting the crystallization.

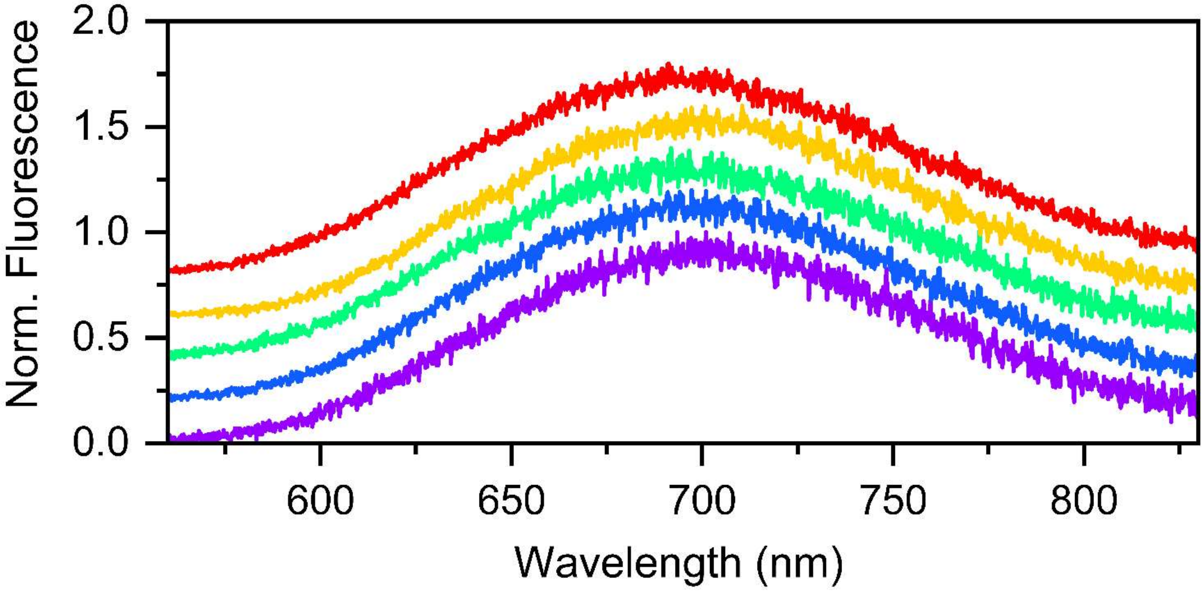

Figure S8: Emission spectra of five different DNA-A_{10}:Ag_{16}NC crystals, recorded with a confocal microscope upon 507.5 nm excitation. The spectra are normalized to the emission maximum and have a constant 0.2 offset for displaying purposes.

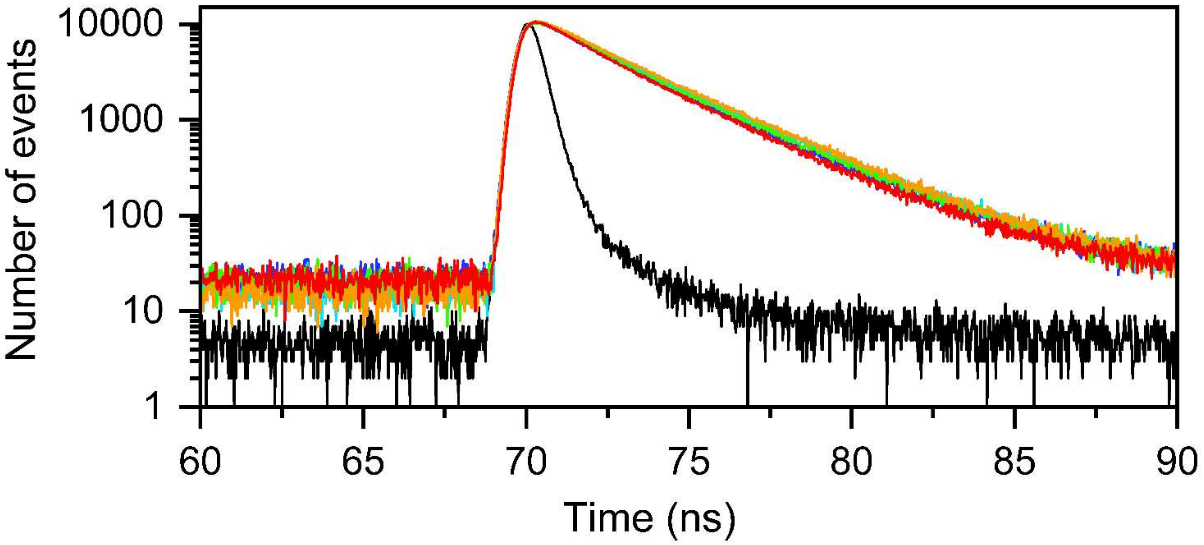

Figure S9: Fluorescence decay time measurements of five different DNA-A_{10}:Ag_{16}NC crystals. The colored curves are the fluorescence decays, while the black curve is the instrument response function (IRF). Every decay was tail-fitted with 2 exponents, and intensity-weighted average decay times of 2.75, 2.85, 2.83, 2.91 and 2.62 ns were found for the individual crystals. The average value (2.79 ns) obtained from these five values is used in the manuscript.

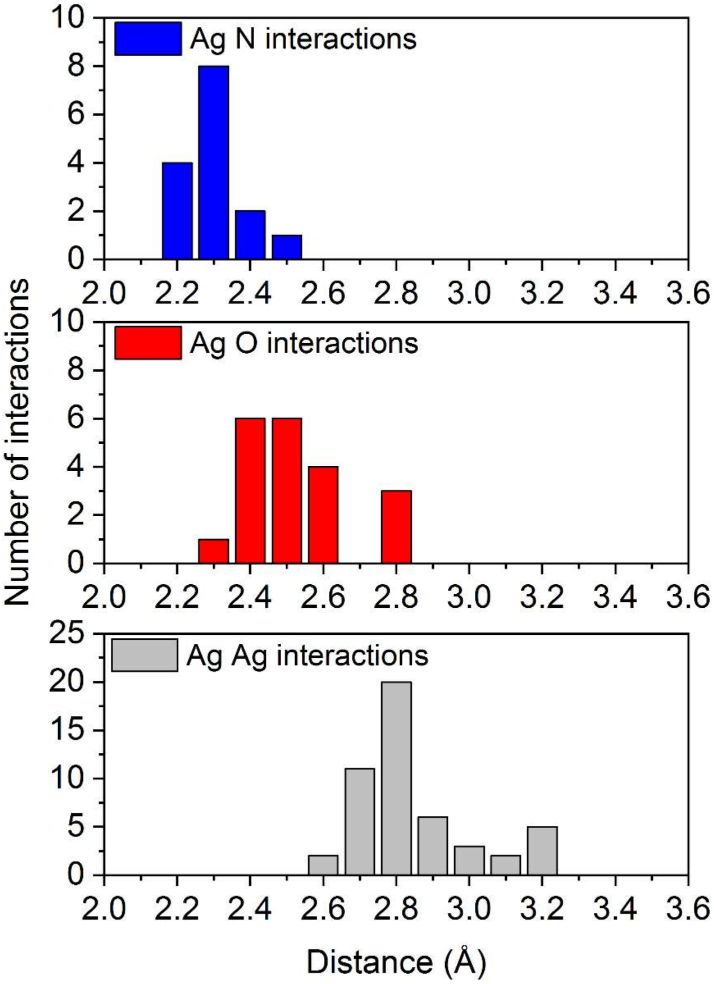

Figure S10: Distance histogram of coordination bonds (Ag-N, Ag-O) and Ag-Ag interactions.

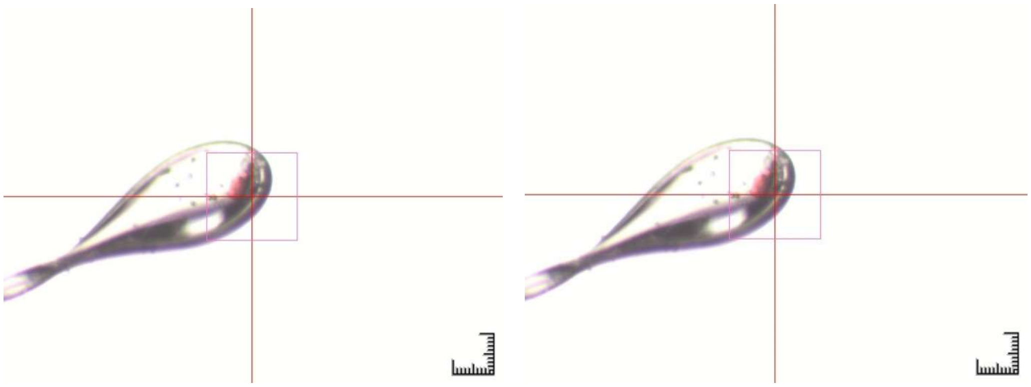

Figure S11. Pictures of a DNA-A_{10}:Ag_{16}NC crystal before (left) and after (right) X-ray data collection from 0 º to 360 º rotation with 0.5 s exposure per 1º oscillation.

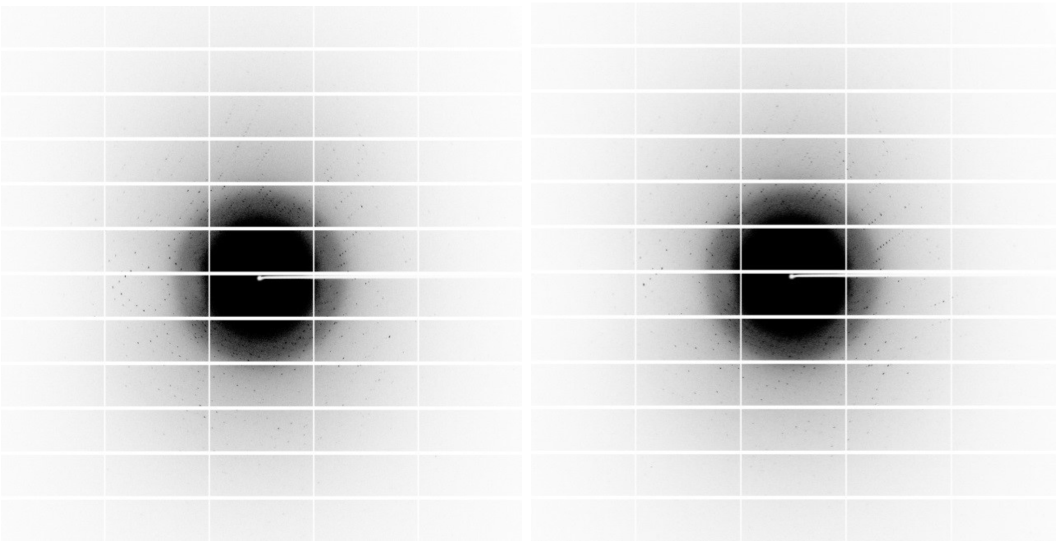

Figure S12. Diffraction images after 0.5 s exposure (left) and 180 s exposure (right). The dataset was taken using 1° oscillation with 0.5 s exposure per frame.

Table S3. Crystal data and statistics of data collection and structure refinement.

Crystal data	
Space group	$P2_1$
Unit cell (Å, °)	a = 24.2, b = 33.7, c = 58.5, β = 98.1
No. of DNA strands in AU [a]	4
No. of Ag atoms in AU [a,1]	32
No. of additional Ag^+ positions in AU [a,2]	4
No. of Ca^{2+} ions in AU [a,1]	2
Data collection	
Beamline	BL-5A in Photon Factory
Wavelength (Å)	1.0
Resolution (Å)	29.1 – 1.1
of the outer shell (Å)	1.15 – 1.1
Unique reflections	67312
Completeness (%)	96.8
in the outer shell (%)	92.5
R_{anom} [b] (%)	5.0
in the outer shell (%)	35.9
Redundancy	3.4
in the outer shell	3.2
$I/\sigma(I)$	16.9
in the outer shell	3.3
Structure refinement	
Resolution range (Å)	29.1 – 1.1
Used reflections	67302
R-factor [c] (%)	8.5
R_{free} [d] (%)	9.6
R.m.s.d. bond length (Å)	0.012
R.m.s.d. bond angles (°)	1.5

[a] Number of molecules, atoms or ions in the asymmetric unit.

[b] $R_{merge} = R_{anom} = 100 \times \Sigma_{hklj}|I_{hklj}(+)-I_{hklj}(-)| / \Sigma_{hklj}[I_{hklj}(+) + I_{hklj}(-)]$.

[c] R-factor = $100 \times \Sigma||F_o| - |F_c|| / \Sigma|F_o|$, where $|F_o|$ and $|F_c|$ are optimally scaled observed and calculated structure factor amplitudes, respectively.

[d] Calculated using a random set containing 10% of observations.

[1] With occupancy 1

[2] With occupancy significantly below 1

References

1. Brouwer, A. M., Standards for photoluminescence quantum yield measurements in solution (IUPAC Technical Report). **2011,** *83* (12), 2213.
2. Bogh, S. A.; Carro-Temboury, M. R.; Cerretani, C.; Swasey, S. M.; Copp, S. M.; Gwinn, E. G.; Vosch, T., Unusually large Stokes shift for a near-infrared emitting DNA-stabilized silver nanocluster. *Methods Appl. Fluoresc.* **2018,** *6* (2), 024004.
